# Is it time for studying real-life debiasing? Evaluation of the effectiveness of an analogical intervention technique

**DOI:** 10.3389/fpsyg.2015.01120

**Published:** 2015-08-04

**Authors:** Balazs Aczel, Bence Bago, Aba Szollosi, Andrei Foldes, Bence Lukacs

**Affiliations:** ^1^Institute of Psychology, Eotvos Lorand UniversityBudapest, Hungary; ^2^Paris Descartes UniversityParis, France; ^3^Corvinus University of BudapestBudapest, Hungary

**Keywords:** judgment and decision making, heuristics and biases, debiasing, analogical training, intervention assessments

## Abstract

The aim of this study was to initiate the exploration of debiasing methods applicable in real-life settings for achieving lasting improvement in decision making competence regarding multiple decision biases. Here, we tested the potentials of the analogical encoding method for decision debiasing. The advantage of this method is that it can foster the transfer from learning abstract principles to improving behavioral performance. For the purpose of the study, we devised an analogical debiasing technique for 10 biases (covariation detection, insensitivity to sample size, base rate neglect, regression to the mean, outcome bias, sunk cost fallacy, framing effect, anchoring bias, overconfidence bias, planning fallacy) and assessed the susceptibility of the participants (*N* = 154) to these biases before and 4 weeks after the training. We also compared the effect of the analogical training to the effect of ‘awareness training’ and a ‘no-training’ control group. Results suggested improved performance of the analogical training group only on tasks where the violations of statistical principles are measured. The interpretation of these findings require further investigation, yet it is possible that analogical training may be the most effective in the case of learning abstract concepts, such as statistical principles, which are otherwise difficult to master. The study encourages a systematic research of debiasing trainings and the development of intervention assessment methods to measure the endurance of behavior change in decision debiasing.

## Introduction

The early observations of a normative-descriptive gap in human judgment and decision making (Tversky and Kahneman, 1974) has given rise to a prolific research field with the central aim of describing how and why human reasoning falls short of logical, economical, or statistical normative ideals. Studied mostly in laboratory situations, people tend to show systematic biases in judgment and decision making tasks (Gilovich et al., 2002). Although the interpretation of these results are not without debate (e.g., Gigerenzer and Todd, 1999; Klein, 1999), a persistent assumption is that people either miss the adequate background knowledge for certain decision problems (Perkins et al., 1993), or they prefer to rely on simple heuristics and strategies that require low cognitive effort, but potentially lead to suboptimal decisions (Kahneman, 2011).

Although these biases and fallacies can impact people’s life to a great degree (Parker and Fischhoff, 2005; Lunn, 2013), our understanding of how to improve human decision making is far from advanced. One factor that might have curbed the enthusiasm of researchers in this area is that the initial debiasing studies showed pessimistic results (Fischhoff, 1981), suggesting a robustness of human decision biases. Nevertheless, one can be hopeful about debiasing if considering that general aptitude correlates positively with normative responses (Larrick et al., 1993; Stanovich and West, 1998), or that studying statistics or economics makes one less likely to succumb to decision biases (Lehman and Nisbett, 1990; Fennema and Perkins, 2008).

### General Debiasing Strategies

In the debiasing research, the identification of the intervention methods mostly follows speculations. An obvious speculation for improving decision making could be that experience would ultimately teach people how to avoid bad decisions, so increasing the amount of experience within a domain should improve the quality of the individual’s decisions (Kagel and Levin, 1986). As intuitive as it sounds, there are several reasons why experience itself will not necessarily debias judgments and decisions. Firstly, people often use feedback only from a subset of events due to unreliable learning environments (Hogarth, 2001) or they themselves bias the encoding and recall of the feedback due to self-serving attributions (Mezulis et al., 2004). The received feedback is often delayed and it is difficult to identify the determinants of the successes and errors (Tversky and Kahneman, 1986). Secondly, people tend to evaluate decisions not by how they were made, but only by their outcomes, which can be misleading in uncertain environments (Baron and Hershey, 1988).

Another common assumption is that improving people’s knowledge about normative rules, such as statistical principles, will have a general effect on the quality of their decisions. To test this, Nisbett et al. (1983) and Fong et al. (1986) initiated an extensive research program and found that by the use of specific cognitive factors, the learning and use of statistical rules may be facilitated within a domain type. However, the transfer of this learning to new domains is rather elusive (Fong and Nisbett, 1991). Critical thinking is also generally assumed to be a potential means to overcome cognitive biases (Baron, 2000). In fact, the aim of critical thinking tests is mostly to assess the person’s ability to avoid biased and erroneous reasoning (Ennis, 1991). Teaching critical thinking, however, is not just a challenging task (Willingham, 2008), but the magnitude of its average effect is surprisingly small (Niu et al., 2013).

A similar meta-strategy for debiasing is the generation of bias awareness (Fischhoff, 1981). The argument of this approach is that knowing about the existence of the bias should reduce its effect (Babcock and Loewenstein, 1997). It was found, however, that knowing about the bias alone is not sufficient; understanding the underlying decision mechanisms has a more direct debiasing effect (Mowen and Gaeth, 1992). Still, general knowledge about the biases without adequate coping skills may fall short of applicability, as was shown in several early debiasing studies (Slovic et al., 1980).

### Specific Debiasing Strategies

Most of the debiasing research has been dedicated to developing and evaluating specific strategies addressing individual biases, such as overconfidence (Renner and Renner, 2001) or sunk cost (Soman, 2001), using a great variety of methods (for a discussion see Mellers and Locke, 2007). As a result, the field of debiasing research became highly fragmented. Arkes (1991) tried to categorize the debiasing methods into three broad categories of biases: strategy-based errors, association-based errors, and psychophysically based errors. He argues that a few general causes are responsible for these biases, and for this reason, they require similar remedies. Strategy-based errors, in which people use inferior strategies, should be decreased by increasing the accountability of the decision maker. Association-based errors, where the initial answer is misleading, should be lowered by instructions. Finally, the psychophysically based errors, such as contrast effect, are to be decreased by altering reference points or reframing the problem. This categorization relies on the assumption that the wide range of biases is the product of a few well-definable underlying factors. Since the work of Arkes (1991), the empirical findings depict a more complex portrait of the taxonomy of decision biases. It is not just that an individual bias can result as an interplay of different cognitive factors (e.g., confirmation bias; Nickerson, 1998), but also, biases previously believed to be unitary (such as the framing or anchoring effect) turned out to be labels for different and dissociating effects (Levin et al., 1998; Epley and Gilovich, 2001).

#### Technological Strategies

The diverse collection of debiasing techniques can be also categorized by their nature. Larrick (2004) suggested grouping these techniques by their technological, motivational, and cognitive aspects. Technological strategies such as using quantitative models (e.g., Dawes and Corrigan, 1974) or checklists (e.g., Hales and Pronovost, 2006) have repeatedly been demonstrated to amend human fallibility. The introduction of these technological solutions is not in the focus of the present paper; however, it is worth mentioning that recent years have witnessed a boom in this area of debiasing. Firstly, clever tricks have been invented to improve the accuracy of our judgments. For example, Herzog and Hertwig (2009) suggest that when we have to guess about something measurable (e.g., the age of someone) we should assume that our first guess is wrong and we should guess again. By this *dialectic bootstrapping* we ‘bracket the truth’ and the average of the two guesses is more likely to be closer to the truth than either of the guesses. Also, asking a few people seems to be enough to use the *wisdom of crowd* to improve the accuracy of our estimate (Larrick et al., 2012). A very different recent approach for dealing with biased decisions is to accept that people are imperfect with their decisions and, instead, we should modify their environment to reduce the chance of errors. Thaler and Sunstein (2008) describe a list of “choice architecture” methods (e.g., social norms, salience, commitment) that can *nudge* people’s decisions in a wiser direction. The advantage of this *Nudge* approach is that it doesn’t rely on the improvement of the people and it has been shown to be a low cost intervention in areas from improving health behavior (e.g., Marteau et al., 2011) to increasing tax compliance (e.g., Castro and Scartascini, 2013).

#### Motivational Strategies

Larrick (2004) suggest that improving the motivation of people represents a separate category of the debiasing techniques. One apparent way to motivate people to perform better is to increase their incentives. Experimental economics relies strongly on the assumption that increased monetary reward induces the subjects to expend more cognitive effort on reflection and calculation, which should ultimately lead to the reduction of errors in performance (Smith and Walker, 1993). A prerequisite for this assumption is that the individual must possess the necessary skills or strategies and only the lack of effort is responsible for their under-performance. In their extensive review, Camerer and Hogarth (1999) found little evidence that people act more rationally at high stakes than at low stakes. Incentives have been found to be effective mostly at tasks where people generally possess the cognitive capital that the work requires (such as clerical work). Decision making tasks, however, are often quite complex or they require the decision maker to recognize when to apply them. In fact, increasing the incentives can also lead to a decrease in performance by applying the wrong strategy with more determination (Hogarth et al., 1991). Although increasing incentives does not serve as a general recipe to improve decisions, they seem to work in certain cases (e.g., Epley, 2004) or they can simply motivate people to learn about more specific debiasing techniques (Camerer and Hogarth, 1999).

People can be motivated to improve their decisions not just by external incentives, but also by social benefits. For example, holding people accountable for their decisions was found to be an effective motivator to decrease certain decision biases (Lerner and Tetlock, 1999). Making favorable impressions or avoiding embarrassment seem to serve as strong social benefits and induce people to use pre-emptive self-criticism before making decisions (Larrick, 2004). A danger in the indiscriminate application of this technique is that it can motivate the decision maker to construct justification for the decision rather than improving the decision process (Shafir and LeBoeuf, 2004).

#### Cognitive Strategies

A third category of debiasing techniques in Larrick’s (2004) system is the various cognitive strategies that serve to change the decision maker’s perception and approach of the different decision problems. Based on the review of 62 articles investigating 72 different debiasing methods, Kaufmann et al. (2010) further categorized these cognitive strategies by their main focus, which can be on (1) structure, (2) perspective or (3) outcome. Many studies have found that decomposing and restructuring the decision-related information have a beneficial influence on the accuracy and correctness of decisions (e.g., Coupey, 1994; Ashton and Kennedy, 2002). The aim of the ‘perspective focus’ strategies are to change the person’s self-centered perspective for the perspective of an outsider or another involved party. This tactic can reduce misprediction about others’ behavior (Faro and Rottenstreich, 2006), which can be especially beneficial in negotiation (Bazerman and Neale, 1982). Taking an outsider’s perspective appears to be especially practical in real-life since the findings show that people tend to avoid the planning fallacy more when they estimate others’ completion time (Buehler et al., 1994). Drawing attention to alternative outcomes has also been found to be a useful tool to reduce the effect of several biases (e.g., Lowe and Reckers, 1994). This technique can effectively counteract people’s tendency to consider only supportive evidence for their hypothesis (Nickerson, 1998). “Consider the opposite” is a similar tactic by asking ourselves how would we know if we were wrong before making our judgments (Gilovich, 1991). This method was found to effectively reduce biases such as overconfidence (Arkes, 1991) or anchoring (Mussweiler et al., 2000). Nevertheless, it can backfire if too much listed counterevidence can similarly bias the decision (Roese, 2004).

### The Challenge of Transfer

From what we have learned about decision debiasing it seems that it is not enough to educate people about the existence of biases and their functionality; they also need to acquire specific debiasing strategies to cope with these challenges. What makes debiasing even more difficult is that decision makers have to recognize the situations in which they need to use the strategies they learned. This task requires transfer from the rule they learned during the training event to the test situation or (preferably) to any analogous real-life situation. The question is how to train people on an abstract rule that they would apply in various relevant situations.

In studies of reasoning, some evidence indicates that practicing only abstract rules can improve performance on specific problems. For example, Fong et al. (1986) found that after training on the law of large numbers where the participants were taught about statistical notions such as sample, population and variability, they were better at reasoning about various uncertainty-related problems, such as slot machines, lotteries, or athletic performance. Similarly, undergraduate and graduate training in psychology and social sciences (Lehman et al., 1988; Lehman and Nisbett, 1990) has been found to increase the students’ ability in reasoning about everyday problems involving uncertainty. Nevertheless, it is hard to assume that these people relied only on their abstract knowledge for the new cases and did not benefit from the concrete examples (e.g., the urn problem demonstrations in the study of Fong et al., 1986) use during the training. Closer examination of these results suggests that it is easier to apply the abstract rules in cases with matching superficial features. For example, Cheng et al. (1986) showed that abstract training of the obligation rule (“If precondition P is satisfied, action A must be taken”) improves performance on Wason’s (1966) four-card problem, but only on those versions of the task where the obligation rule could be used in the task. Fong and Nisbett (1991) taught their participants about the law of large numbers in either one of two domains and they were tested on both domains. Although immediately after the training, they found no effect of domain, 2 weeks later the participants could perform better in the domain they were taught in. In their summary, Smith et al. (1992) suggest that in situations where more than one mechanism is involved, reasoning might rely on hybrid instance-rule mechanisms. Therefore, superficial similarity between the learned instance and the target case can facilitate rule-application. This suggestion is in accord with studies of problem solving where it is assumed that a major cause of failures to transfer the relevant rule to analogous situations is the greater attention people pay to the salient and superficial details at the time of learning and that they will apply the learned principles in the test situation to the degree that it shares those contextual features (Holyoak and Koh, 1987; Ross, 1987).

Thompson et al. (2000) and Gentner et al. (2003) introduced a method to foster this analogical transfer. The idea behind the method is that people can better encode the principles if they discover them themselves. They suggest that schema abstraction can be promoted by asking people to find similarities in superficially different cases. According to the structure-mapping theory (Gentner, 1983), making comparisons should highlight the structural similarities between examples with different surface features. This discovered common relation can be better encoded and retrieved in the future. A further advantage of this practice is that it also helps the learner identify the relevant aspects of the examples. This analogical encoding method was tested primarily in negotiation skill training. Thompson et al. (2000), for example, found that when management students learned about bargaining principles through comparisons they were nearly three times more likely to transfer the principles to actual bargaining situations than those who only read the cases. Beside several negotiation studies (Loewenstein et al., 2003; Gentner et al., 2004; Moran et al., 2004) analogical encoding was also found to help the debiasing of the robust Acquiring Company Problem (Idson et al., 2004). In this task (for detailed description see Samuelson and Bazerman, 1985), the decision maker has to decide what price an acquirer company should offer for a target company if the acquirer knows that it will be worth 50% more in their possession, although only the target company knows the current worth exactly. It is a general finding that fewer than 10% of the participants realize that the best choice is $0 (Bazerman, 2005). A protocol analysis conducted on this task (Tor and Bazerman, 2003) showed that the main reason why people fail this task is that they ignore the rules of the game, that the target company has unique information as well as they ignore the decisions of the other parties. Idson et al. (2004) managed to improve performance on this task by requiring the participants to understand the differences between two different tasks prior solving the Acquiring Company Problem. The Monty Hall Game (Nalebuff, 1987) and the Multiparty Ultimatum Game (Messick et al., 1997) tasks are seemingly unrelated, yet discovering their structural differences can train the participants to focus on the key features necessary to solve the Acquiring Company Problem. Analogical processing, therefore, appears to be effective in overcoming failures in a variety of tasks. Interestingly, the effect seems to be stronger when the compared cases are more diverse (Moran et al., 2008) and it facilitates not just transfer, but also the retrieval of analogical matches stored in memory (Gentner et al., 2009). These results suggest that the facilitation of analogical processing should be further explored in improving decision making.

### The Aim of Debiasing Research

The central aim of the prescriptive approach of decision making is to identify those tools and methods that can be applied to improve the quality of decisions. Decision making is a central determinant of quality of life, interpersonal relations, economics and societal welfare. Therefore, the final aim of decision science, and especially the debiasing approach, should be to promote the improvement of decision skills and rational behavior in everyday life. Despite the high number of studies in this approach, two important aspects have received insufficient attention. Firstly, most studies concentrate on immediate changes in decision making and the long-term effect of the training methods are rarely assessed. For this reason, our knowledge is rather scarce about what elements can achieve lasting change in decision skills. Also, we do not know how to achieve acceptable cost-benefit ratio of these interventions (Arkes, 1991). Secondly, the applied training techniques are often applicable only in laboratory settings, and miss those motivational and interactive features that could make them transferable to and applicable in non-academic settings.

From this aspect, the challenge of debiasing is not just to identify those techniques and strategies that are potentially effective in achieving lasting improvement, but also to connect the interventions to the everyday world of the individuals. The problem of the field is the fundamental conflict of interest between the researcher and the potential practitioner with respect to the complexity of the training methods. From the researcher’s perspective, the applied method should be reducible to one controlled variable, studied in laboratory settings. The training should focus on a single decision bias in a particular domain. From the practitioner’s perspective, the training methods should promise enduring improvement in various areas of decision making. The interventions should be applicable in different environments and they should match the interest, motivation, capacity and attention of the individuals.

Although it is an understandable aim of the researcher to minimize the complexity of the experiments to sustain the conceptual clarity of the measurements, real-life debiasing methods also need evidence-based development. Clinical therapies show us that intervention methods can be both broadly applicable and empirically tested. Debiasing would greatly benefit from a similar exploration to learn what works and what does not. Sporadic individual studies will not be enough to achieve the societal aim of debiasing, and so we argue that for this a wider research program is needed.

The goal of this research is to initiate the exploration of the potential debiasing training techniques for lasting improvement for multiple biases. In this study, we tested the duration of the effect of an analogical debiasing method. Toward this aim, we developed an analogical training for 10 decision biases based on the principles of the analogical transfer technique of Loewenstein et al. (2003). The structure of this development followed Lewin’s (1947) recommendation: Unfreezing, Change, Refreezing. The aim of Unfreezing is to make the decision makers realize that their current intuitive strategies are flawed. For this, we asked the participants to answer questions in an assessment test after which the software generated a report showing which of the situational questions they failed. The Change phase was preceded by analogical sensitization training where the participants learned to recognize the structural similarities between superficially different cases. The Change phase always started with situational tasks or examples of people committing the given bias. Their task was to discover the essence between or principle behind the examples. Once they discovered the principle, the trainer provided a description of the functionality of the bias. Then the participants had to recall autobiographical memories about committing the bias. Next, the trainer provided coping strategies for similar situations and for Refreezing the participants had to predict when they will use the newly learned strategy. Four weeks after the training the participants were tested again with a different version of the survey than the one they filled out before the training. The empirical aim of this project was to compare the effect of this analogical training to the cases where people received only awareness training or where they did not receive training at all.

## Materials and Methods

### Participants

One fifty-four (118 female) Hungarian university students took part in the experiment (*M* = 21.73 years, SD = 3.61) for course credit. After obtaining informed consent they were randomly assigned to one of the three groups: two experimental groups (*n* = 50 each) and one control group (*n* = 54). The research was approved by the institutional ethics committee of Eotvos Lorand University, Hungary.

### Design and Procedures

The participants of the two experimental groups took part in 3-h long debiasing training in group sessions with 6–17 participants in each session (a total of nine sessions were conducted in the whole experiment). All sessions followed the same structure for both groups with the same main phases: (1) awareness training, (2) analogical sensitization and (3) analogical training. **Table 1** gives a summary of the design of the experiment; the different training phases are described in more detail in the Section “Materials.”

**Table 1:** Overview of the experimental design.

	Group 1 (*n* = 50)	Group 2 (*n* = 50)	Control group (*n* = 54)
Test (before the training)	An online questionnaire for assessing the susceptibility to decision biases.		
Awareness training	Training for: - Outcome bias; - Sunk cost fallacy; - Base rate neglect; - Insensitivity to sample size; - Regression to the mean; - Covariation detection.	Training for: - Framing effect; - Anchoring bias; - Overconfidence bias; - Planning fallacy.	No training
Analogical sensitization	Familiarizing participants with analogical thinking.		
Analogical training	Training for: - Framing effect; - Anchoring bias; - Overconfidence bias; - Planning fallacy.	Training for: - Outcome bias; - Sunk cost fallacy; - Base rate neglect; - Insensitivity to sample size; - Regression to the mean; - Covariation detection.	
Test (after the training)	An online questionnaire for assessing the susceptibility to decision biases.		

Although both experimental groups received debiasing training for a total of 10 biases and fallacies, the experimental conditions were crossed between them: while one group received *awareness training* for six of the biases (Covariation detection, Insensitivity to sample size, Base rate neglect, Regression to the mean, Outcome bias, Sunk cost fallacy) and received *analogical training* for the remaining four biases (Framing effect, Anchoring bias, Overconfidence bias, Planning fallacy), the other group received them in inverse (analogical training for the same six biases and awareness training for the remaining four). The explanations of these biases are provided in Supplementary Table S1. We trained Covariation detection, Insensitivity to sample size, Regression to the mean and Base rate neglect together as ‘statistical biases’ based on the similarity of the skills needed to overcome them (the usage of statistical rules). It is important to emphasize that for these biases participants did not receive training separately: the biases were presented together for both the awareness and the analogical training groups.

To assess susceptibility to these decision biases, the participants were required to complete an online questionnaire 1 day before the training sessions. Four weeks after the sessions, the participants were asked to complete an altered version of the online test within 3 days. The control group received no training; instead, they were only required to complete the two versions of the online questionnaire with the same time delay between the two parts as the experimental groups.

### Materials

#### The Online Questionnaire

The susceptibility of the participants to nine biases was assessed on an online questionnaire (Supplementary Table S1). The questionnaire was developed as a part of one of our previous, unpublished studies. As the planning fallacy could not be measured the same way as the other biases, the effectiveness of the trainings for the planning fallacy was not assessed. Each of the other biases was measured by one task in the questionnaires. The tasks were adapted from the heuristics and biases literature with modifications necessary for the requirements of our study. For each question, four possible answer options were presented with only one being the normatively correct one. Participants scored either 1 for correct answers or 0 for incorrect answers. In case of Overconfidence, the perceived accuracy score of the participant was subtracted from their real accuracy score. To increase the motivation of the participants to give the right answer, the wording of the questions was modified to place the participant in the role of the agent responsible for making the best possible decision in the given critical situation.

Two versions of the questionnaire were used: one before the experiment and one after. Only the described decisional situations were different in the two versions, the underlying structure of the tasks was retained. After solving the questionnaires, the participants received a summary table of their performance on each task, with a brief description of that task. The completion of each survey took ~25 min. Time pressure was also applied on each question (30–60 s). In the second survey, besides the heuristics and biases tasks, we also asked the participants about how much they utilized the learned techniques following the training.

#### The Training Sessions

The phases of the training were connected by a continuous Power Point presentation, with a brief break after the awareness training phase. Participants took an active role in the analogical sensitization and analogical phases, while the awareness phase followed a presentation. **Table 2** gives a comprehensive summary of the different phases.

**Table 2:** A detailed overview of the training phases.

Phase	Sub phase	Task	Goal	Duration
Awareness training	Introduction	No active participation	Familiarizing participants with the aim and duration of the training followed by short presentation on intuitive decision making in general.	~15 min
	Presentation		Familiarizing participants with specific biases using illustrative examples and coping techniques for each bias.	~45–60 min
Analogical sensitization	Workshop	Pairing different scenarios by similarity to understand structural and surface analogies, followed by tasks involving the recognition of structural similarity.	Familiarizing participants with the difference between surface and structural similarity and practicing analogical thinking	~10 min
Analogical training	Analogical encoding	Discovering and discussing structural similarity in vignettes describing new biases.	Developing new schemas from examples for future recognition.	~120 min
	Presentation	No active participation	Describing the specific biases to participants.	
	Pattern recognition	Construction or recall of scenarios structurally similar to the example.	Recognizing the bias-specific patterns in everyday life.	
	Action plan	Participants learn bias-specific action plans that help them avoid biased decision making.	Participants understand what to do when they encounter the newly learned patterns.	

#### The Awareness Training

In the awareness training phase a short general introduction of heuristics and biases was followed by the presentation of each bias. In the introductory phase, besides giving information about the duration and the aim of the training, to ‘unfreeze’ the participants the flaws of intuitive decision making were demonstrated by several examples, with the conclusion that in real life our intuition can often misguide us. Participants also received a feedback presentation on how people make mistakes on tests similar to theirs.

The presentation of the biases consisted of three parts: a real life example, an explanation of the bias and the techniques to avoid the bias. Participants in this phase were only presented with the underlying principles and real-life examples of each bias. The different examples and techniques for each bias are detailed in Supplementary Table S2.

#### The Analogical Sensitization

In the analogical sensitization phase pattern recognition was facilitated by two tasks. In the first task (adapted from Gick and Holyoak, 1980), participants were asked to pair up four short scenarios without explicitly telling them the difference between the concepts of structural and surface analogies. In order to understand the difference between these two types of analogies, this pairing task was followed by a group discussion of the solutions. The second task was an integrative bargaining task based on Follett’s (1940) orange-peel example. The participants were expected to reach a trade-off in a negotiation situation, after hearing about a solution in an analogous story. This again was followed by a group discussion which aimed to ascertain that all participants correctly understood the difference between the two types of analogies.

#### The Analogical Training

In the analogical training phase, participants were trained on each bias consecutively following the same structure: at first, groups of two or three were asked to accomplish different tasks with the aim of detecting structural similarities between stories or situations containing the same bias. After discussing their interpretation with the whole group, the participants received the same presentation on the normative principles along with examples of the biases, as did the other experimental group in the awareness phase. After this, participants were asked to recollect memories or try to imagine future occasions when they might commit the bias in their everyday life. Following this, they were first asked to suggest strategies to avoid the biases, and then they were presented with specific research-based coping techniques. Finally, the participants had to discuss how they would utilize these techniques in real life.

The examples and the avoidance techniques of the biases used in both the awareness and analogical conditions were the same (see Supplementary Table S2). For the description of the specific training tasks, see Supplementary Table S3.

## Results

### Bias Assessments

The initial assessment of the performance on the pre-test questionnaire showed that participants were generally susceptible to the measured biases (**Table 3**). Three of the statistical biases (covariation detection, regression to the mean, insensitivity to sample size) showed the weakest performance, and participants resisted the anchoring bias the most.

**Table 3:** Percentage of correct responses for each bias.

Bias	Percentage of correct responses (%)
Anchoring bias	63.96
Framing effect	50.00
Outcome bias	44.48
Base rate neglect	44.00
Sunk cost fallacy	41.56
Overconfidence bias	35.06
Covariation detection	27.27
Regression to the mean	20.78
Insensitivity to sample size	16.23

### Statistical Analysis

Since the participants answered an online questionnaire before and after the treatment, both within-subjects (pre- and post-training tests) and between-subjects (awareness training, analogical training, control) measures were collected throughout the experiment. As the two versions of the test may not be equally sensitive, accuracy was used as a criterion variable in the models. To control for statistical noise (e.g., cohort-effects), hierarchical linear logistic mixed effect models were used with the lme4 (Bates et al., 2014) and nlme (Pinheiro et al., 2014) packages in R. Fixed effects were entered hierarchically to the model: first test-score differences (the differences between pre-training and post-training test-scores), then experimental manipulation (analogical, awareness or control), and finally the interaction between them. The participants were entered as random effects to the models.

If the manipulation had an effect, the inclusion of the interaction term would significantly improve model fit. This would mean that the observed variance in accuracy could not only be the result of the different sensitivity of the two tests or of random noise, but it was affected by the experimental manipulation. To assess the order of the differences (i.e., whether the analogical training was more effective than the awareness training), the different conditions were compared hierarchically stepwise for each bias: first, the awareness and the control conditions were compared, to see whether the awareness training had any effect; then the analogical condition was compared to the awareness condition, to determine whether it brought any further improvement.

#### Effects for the Separate Biases

The analyses of the individual biases revealed that 4 weeks after the training, the participants showed improved decision making only for the composite score of statistical biases (which was calculated as the sum of the scores of the Insensitivity to sample size, the Base rate neglect, the Regression to the mean, and the Covariation detection tasks), χ^2^(9) = 7.58, *p* = 0.02. Participants did not show significant improvement for the Framing effect, χ^2^(7) = 1.05, *p* = 0.59, the Anchoring bias, χ^2^(7) = 0.94, *p* = 0.62, the Sunk cost fallacy, χ^2^(7) = 3.19, *p* = 0.2, the Outcome bias, χ^2^(7) = 2.71, *p* = 0.26, and the Overconfidence bias, χ^2^(7) = 2.29, *p* = 0.32.

**FIGURE 1:**
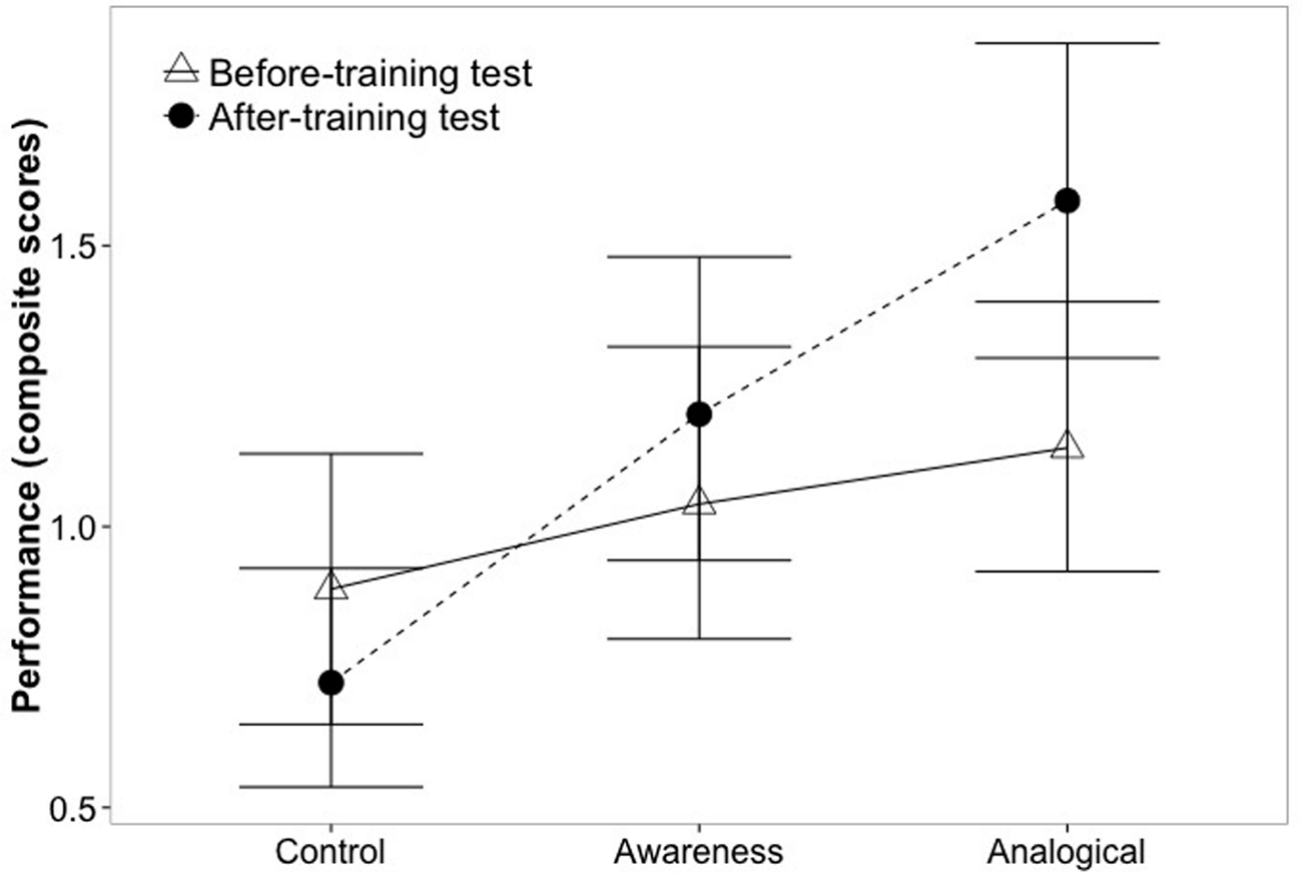
**Performance in the different conditions for the statistical biases.** Means and 95% bootstrapped confidence intervals for pre- and post-training tests for each condition are presented.

In the case of the statistical biases the significant 3 × 2 interaction indicates changes in the pattern of the development through training conditions (from the control to the analogical condition). This effect is demonstrated on **Figure 1** where an increase can be observed regarding the performance between pre- and post-training scores of statistical biases. Participants in the control condition showed the lowest performance, while this value was the highest for the analogical condition. The results can be interpreted more easily with Cohen’s *d* values between the pre- and the post-training test means in every condition; the smallest Cohen’s *d* can be observed in the control group, while the highest can be observed in the analogical training group (descriptive statistics and Cohen’s *d* effect sizes are available in the Supplementary Material). This trend is reflected in the finding that only the analogical training group performed significantly better than the control group, *b* = 0.61, *t*(151) = -2.75, *p* = 0.007, η^2^ = 0.22. However, the difference between the two training groups was not significant, *b* = 0.28, *t*(151) = 1.25, *p* = 0.21, η^2^ = 0.1. Nevertheless, participants in the awareness group did not show significant performance increase compared to the control group, *b* = 0.33, *t*(151) = -1.48, *p* = 0.14, η^2^ = 0.12.

**Figure 1** illustrates the performance on the different conditions for statistical biases. An increase can be observed regarding the performance between pre- and post-training scores of statistical biases; participants in the control condition had the lowest performance change, while in the analogical condition an increase in performance can be observed. The results can be interpreted more easily with Cohen’s *d* values between the pre- and the post-training test means in every condition; the smallest Cohen’s *d* can be observed in the control group, while the highest can be observed in the analogical training group. This trend (the difference between Cohen’s *d* among experimental conditions) represents the effect of the analogical training (descriptive statistics and Cohen’s *d* effect sizes are available in the Supplementary Material).

**FIGURE 2:**
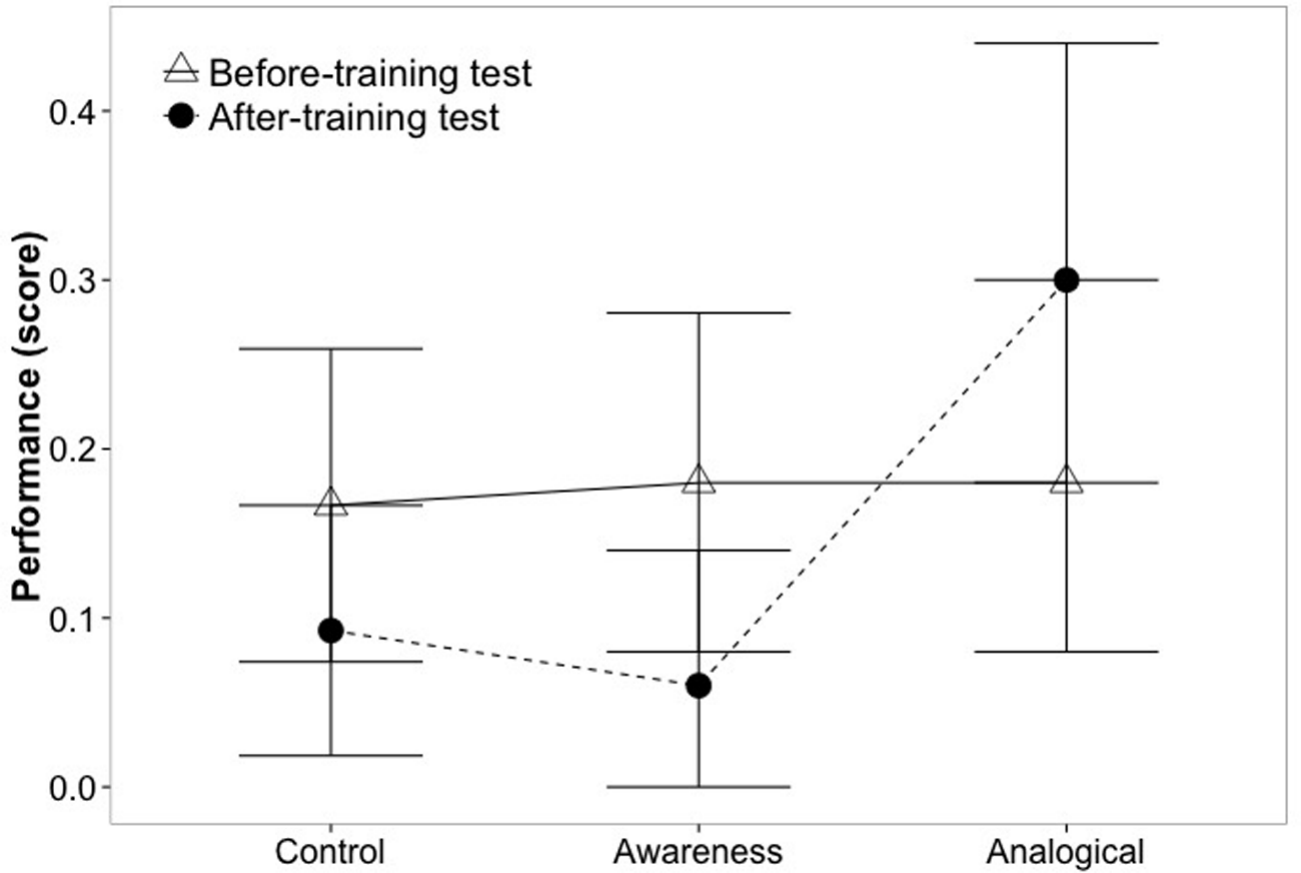
**Performance in the different conditions for Insensitivity to sample size.** Means and 95% bootstrapped confidence intervals for pre- and post-training tests for each condition are presented.

In the individual analysis of the statistical biases, we found that the inclusion of the interactional term significantly improved model fit only for the Insensitivity to sample size, χ^2^(7) = 7.3, *p* = 0.03. More specifically, here the analogical training significantly differed from the awareness group; *b* = 2.18, *Z* = 2.36, *p* = 0.02, while the awareness training did not show any difference from the control group, *b* = 0.59, *Z* = 0.62, *p* = 0.54. The performance on the different conditions is illustrated in **Figure 2** for the Insensitivity to sample size, showing a similar trend to the composite of the statistical biases.

#### Reported Effect of the Training for Real Life Decisions

At the end of the second questionnaire, participants of the experimental conditions were asked whether they made any decisions differently in their everyday life after the training using the learned coping strategies. All together 46.15% of the participants reported ‘yes’ to this question. With respect to the statistical biases, 58% of the participants who received analogical training for the statistical biases reported ‘yes’ and only 32.43% of participants who received only awareness training for these biases chose the ‘yes’ option.

## Discussion

In this study, we present a training method that attempts to utilize analogical encoding (Gentner et al., 2004) in order to explore its potentials for lasting decision making debiasing. The method was tested in the case of 10 well-known decision biases and the efficiency of the analogical training was compared to traditional awareness training and a ‘no-training’ control group. To measure the efficiency of the training methods, we assessed the susceptibility of the participants to the biases after the training, and to control for cohort-effects we measured the baseline bias-susceptibility of the participants prior the training. The results indicated that the improvement achieved by the training was detectable for certain biases even 4 weeks after training. We found that a group of biases that we call statistical biases, especially on the Insensitivity to sample size, benefited the most from our training. Yet, for the other biases, we could not detect improvement after either the awareness, or the analogical training.

It is not surprising that the debiasing of the statistical biases benefitted the most from the analogical training since the main advantage of building on analogical processing is that it can decrease the difficulty that people encounter when they try to encode or apply abstract rules (such as statistical principles). Transfer of trained statistical principles have shown success previously with non-bias tasks (Nisbett et al., 1983; Fong and Nisbett, 1991), and training especially increased everyday inductive reasoning when it was built on people’s intuitive statistical knowledge (Nisbett et al., 1983). Various empirical studies suggest, however, that abstract rules are more effectively trained when they are presented in more intuitive representations (such as frequencies instead of probabilities, e.g., Gigerenzer and Hoffrage, 1995); pictorial displays instead of mere text (Galesic et al., 2009); or pragmatic rules instead of pure syntactic rules (Cheng et al., 1986).

Our results also resonate with the suggestion of De Neys and Bonnefon (2013) that thinking biases can occur at different points in the reasoning process. According to their account, a bias can be the result of storage failure, monitoring failure or inhibition failure. The authors argue that most biases in reasoning can be connected to inhibition failure, a later stage of the reasoning process. The framework of Stanovich and West (2008) is more specific about the different paths that can lead decision makers to follow a heuristic response instead of the normative response. Here, the first question again is if the procedures and declarative knowledge (mindware) are available during the process to override the heuristic answer. Failures due to inhibition would come only at a later stage of the process. Based on these accounts, it is possible that the biases relying on statistical principles represent more storage or mindware problems compared to the other biases we measured. In this sense, the analogical training method should be used specifically in cases of mindware problems.

It was, however, surprising to find that 4 weeks after the intervention only limited improvement is sustained from an intensive debiasing training. Most previous debiasing studies measured the degree of improvement in the same session with the intervention (e.g., Larrick et al., 1990; Clarkson et al., 2002; Cheng and Wu, 2010), rarely exploring whether the training achieved an enduring change in the decision maker’s behavior (Fong and Nisbett, 1991). It may be useful if further research of debiasing methods would not be limited to the detection of the immediate debiasing effects, but would more thoroughly explore the endurance of the acquired skills as well. To measure the real-life changes the training achieved, we used a single question assessment. However, to assess the improvement of people’s real-world decision making competence a more thorough test would be necessary. The Decision Outcome Inventory (Parker and Fischhoff, 2005), for example, has been developed to survey real-world decision outcomes which has been found to correlate with tests of decision making competence (Bruine de Bruin et al., 2007; Parker et al., 2015). Although the test typically explores people’s decision outcomes for the previous 10-years frame, it would be practical to devise a version of the test for a much shorter time-frame for assessing the endurance of the acquired skills of decision competence.

While our empirical results require further validation, the main contribution of this paper is the new debiasing method and the training assessment technique it provides. The development of an analogical training method for a greater variety of decision biases shed light on several questions that might desire more attention in the debiasing approach. As discussed in the Introduction, debiasing decision making became a vexing question as simply explaining the existence of biases or providing coping strategies do not seem to significantly improve the quality of decisions beyond the narrow focus of the training or the training situation. One reason for this difficulty may be that people show resistance to being debiased (Arkes, 2003), as they prefer to believe that the decisions they make are generally good and beneficial. This self-image is supported by the well-known self-serving attribution and selective autobiographical processes (Mezulis et al., 2004). On the other hand, when compliance with the rules is induced by reward people may show better performance in the testing situation, but without internalization and voluntary adoption the intended change will not become the behavioral repertoire of the person (Kaplan et al., 2001). From this aspect, it seems that prior debiasing training might have missed putting sufficient focus on what Lewin (1947) called the Unfreezing stage of change. This first step of influencing people to change involves methods to make them understand that change is necessary. In developing our training, we tried to give special emphasis to this phase and made sure that the participants gain experience about how much their judgment can go wrong and to realize how much they could improve the quality of their decisions. Eliciting motivation to change in the participants is especially crucial as recent reviews suggest that the introduction of the various decision aids to organizations results in limited change (McLean and Antony, 2014).

Lewin’s (1947) second stage, Change, was the main focus of the development of this debiasing method. To facilitate the process of change, we chose to make the participants work in groups during the training as individuals are more likely to be influenced by the behavior of their peers and change in this setting (Greenbank and Hepworth, 2008). Considering the method of the training, we argued that to achieve effective behavior change, the decision maker has to be able to recognize those situations and environmental patterns where they have to try to avoid the traps of decision biases. To foster the recognition of structural similarities between superficially different cases of the same bias, the analogical encoding method (Gentner et al., 2004) seems to be the most promising technique. Among the many advantages of this method, it can be used to help decision makers integrate the learned coping techniques into their everyday life by connecting them to their facilitated autobiographical memories (Gentner et al., 2009). In Lewin’s (1947) terminology, the role of this Refreezing stage is to sustain and stabilize the changes that have been made. In our study, we only retested the participants 4 weeks after the training, as our primary interest was to see which of the trained skills resisted attenuation.

This analogical transfer method has interesting resemblance to the case-based reasoning (CBR) approach (Richter and Aamodt, 2005). CBR is based on human problem solving research (Schank, 1983), but mostly applied in artificial intelligence solutions (Watson, 1998). The idea behind the approach is that the human mind is more prone to capture knowledge through specific experiences than via learning abstract rules (Schank, 1999). At problem solving, we rarely recall abstract rules. Rather we try to retrieve the most analogous case we solved before and we adapt those methods to our present case (Lopez De Mantaras et al., 2005). The model of CBR has inspired not just computer-based systems, but also education techniques (Kolodner et al., 2005). These techniques aim to build on analogical reasoning processes in the encoding, retrieval, and adaptation of new information (Kolodner, 1997). It seems that in the context of solving real-world problems, case-based learning is more effective than simple presentation of abstract rules (Kolodner et al., 2003), while abstract principles can be better taught through cases. Jacobson et al. (2012), for example, showed that decision making competence was improved along with academic learning when decision training was integrated in history courses. It is possible that memorable cases of critical decisions helped the student – via analogical encoding – identify situations in which to apply the acquired knowledge. These emerging techniques allow us to take more advantage of the analogical techniques when abstract principles are to be educated in decision debiasing.

Since this analogical debiasing training has been only tangentially studied in relation to the main decision biases and fallacies, we included a wider range of them in this study. Investigating debiasing techniques for multiple biases has the advantage that the corrective effect of the techniques can be compared among the biases and it can lead to the identification of which techniques should be used for the different biases. This assessment method requires that in a multiple choice test the chance level of correct responses are equal for each bias. To satisfy this methodological need we had to use a bias-test where the response mode is the same for all tasks. In our tests, the participants had to choose between four options where only one option was considered the correct response and choosing any of the other three options indicated susceptibility to the specific bias.

A further novelty of our analysis was that we controlled for the baseline differences between the experimental and control groups. Although our participants had been randomly allocated to one of the three groups, individual differences independent of our manipulation can bring noise to the analysis. To avoid this, we introduced a baseline test prior to the training, by which we could concentrate our analysis to the improvement that the intervention achieved from pre-test to post-test.

### Limitations

This study is an early step in discovering the possible advantages of the analogical encoding method for decision debiasing and as such, it comes with limitations where conclusions are drawn. As in the case of any interactive intervention, the experimental variables cannot be entirely controlled due to the practical nature of the training. Consequently, alternative explanations of the effect cannot be completely excluded. Although our results suggest that the analogical training is effective on statistical biases, and when compared to the awareness training almost twice as many participants reported that they tried to apply the learned coping strategies in their everyday life between the training and the second test, this effect is not necessarily the result of the technique itself. The analogical training took somewhat more time than the awareness training, so it is not impossible that spending more time with one bias resulted in more sustainable knowledge of it. In the same vein, it cannot be concluded from the data how much the interactivity and not the analogical nature of the training is responsible for the debiasing effect. Within the analogical training, it would be interesting to know whether the improvement was due to the recognition of structural or the surface similarities between the training and the test examples. It is possible that training on certain biases had a carry-over effect to the training of other biases. Similarly, it is also possible that the trainings had a more general corrective effect on the participants’ decision making than we managed to measure, but that it faded away by the time of our test 4 weeks after the training. People’s susceptibility to the individual biases may be better measured by including more items in the questionnaire and by using more sophisticated questionnaires for the assessment of the changes the training achieves in their life. Further research is needed to explore the potentials of the analogical encoding method for decision debiasing and identify the mediating mechanisms and moderating factors.

## Conclusion

Although the results of the analogical reasoning research were taken as a “tremendous promise” (Bazerman, 2005) for improving decision making, the efficiency of the method has been tested almost only on negotiation strategy training. Nevertheless, this technique, which assists the decision makers to learn and apply abstract principles in structurally similar situations, provides a complementing response to several shortcomings of the commonly used debiasing techniques. This initiative offers some methodological support for a systematic research that can hopefully provide enduring techniques for improving decision making, a chief limitation of the field of behavioral research.

## Further Thoughts

To amend the present hiatus of the debiasing approach of decision science, we believe that a systematic research program should explore the necessary components of those training methods that can achieve long-lasting improvement in decision skills related to a wider array of cognitive biases. Such a program should study the efficiency of both general and specific debiasing techniques. General skills such as critical thinking, need for cognition, analogical thinking, pattern recognition or statistical knowledge have been shown to increase the immunity for bias susceptibility. How to achieve lasting improvement in these skills related to real-life problem solving is a necessary question to answer. Regarding bias-specific debiasing techniques, the challenge is to implement the laboratory findings into practical and testable training solutions. To answer this question, we could benefit from a general overview of the evidence for successful debiasing regarding specific biases. The determinants of ‘refreezing’ is also an open question for further research. To explore this question, attempts similar to our present work are needed in order to develop a methodology for measuring the efficiency and duration of the applied debiasing techniques.

## Conflict of Interest Statement

The authors declare that the research was conducted in the absence of any commercial or financial relationships that could be construed as a potential conflict of interest.
