# Supplementary material for: Is it time for studying real-life debiasing? Evaluation of the effectiveness of an analogical intervention technique
Source: Front Psychol. 2015 Aug 4;6:1120. doi: 10.3389/fpsyg.2015.01120 (PMC4523707; doi:10.3389/fpsyg.2015.01120)
Supplement: Supplementary file 1 [file Data_Sheet_1.DOCX]

***Supplementary Material***

**Is it time for studying real-life debiasing? Evaluation of the effectiveness of an analogical debiasing intervention technique**

**Balazs Aczel^1^*, Bence Bago^2^, Andrei Foldes^1^, Aba Szollosi^1^, Bence Lukacs^3^**

^1^Institute of Psychology, Department of Affective Psychology, Eotvos Lorand University, Budapest, Hungary

^2^Paris Descartes University, Paris, France

^3^Corvinus University of Budapest, Budapest, Hungary

*** Correspondence:** Balazs Aczel, Institute of Psychology, Department of Affective Psychology, Eotvos Lorand University, Izabella u. 46, Budapest, 1064, Hungary

aczel.balazs@ppk.elte.hu

1. **Supplementary Figures and Tables**

## Supplementary Tables - assessment tests

In the two assessment tests, participants on most tasks were required to choose between four answer options where only one of the options was the correct answer. For all bias tasks the chance level of giving correct answer was kept at 25 %. For those biases which can be measured by only two questions (outcome bias, anchoring bias, and framing bias), the two items of the questions were separated within the questionnaire as widely as possible. Correct answers were scored as 1, while incorrect answers were scored as 0. Table 1 describes the specific questions we used for our pre- and post-training tests. The test questions were developed as part of one of our previous unpublished studies.

**Supplementary Table 1. Tasks and questions on the assessments tests.**

| **Bias**  (Description; Source) | **Pre-training tasks** | **Post-training tasks** |
| --- | --- | --- |
| **Outcome bias**  - refers to the tendency of people to evaluate quality of decisions based on their outcome  (Tasks adapted from Baron and Hershey, 1988) | *Task/1*  A biotechnology company is considering the development of a new, innovative technology. If the new technology is successfully introduced to the market, the investment will have a high rate of return. However, experts consider the investment very risky, because the company has to take out a large loan to cover development costs. According to the analysts, there is a 10% chance that the project will fail and the whole company will go bankrupt. The management decided to invest in the development, and the project was successful.  *Task/2*  The management of AeroWings Airline is considering starting a space tourism project. If the project is successful, the investment will have a high rate of return, but experts consider the investment very risky, because the financial burden of the project is very serious. According to analysts, there is a 10% chance that the project fails and the whole company goes bankrupt. The company decided to invest in the development, but the project did not turn out to be successful, and the airline went bankrupt because of loans.  *Answer options for both tasks*  Please evaluate the decision of the company on a 4-item scale.  (1 - Definitely not a good decision; 4 - Definitely a good decision)  *Correct answer*  Consistent choices on the 2 tasks. | *Task/1*  One of your acquaintances told you about an interesting investment opportunity. Based on reliable economic analyses, there is a 90% chance that you make an outstandingly high return on this investment. You could only enter into the investment by risking a large amount of money. You decided to enter the investment. The business was successful, and you made a high return.  *Task/2*  You are the owner and the business manager of a small firm. You are invited to a tender. Winning the tender would guarantee sales returns and outstandingly high profit for your firm during the coming years. Applying for the tender, however, requires a lot of money, so you can expect serious losses if the firm loses the tender. Based on experts' forecast, there is a 90% chance that you win the tender. You decided to apply for the tender. You lost the tender, and the company suffered serious losses.  *Answer options for both tasks*  Please evaluate the decision of the company on a 4-item scale.  (1 - Definitely not a good decision; 4 - Definitely a good decision)  *Correct answer*  Consistent choices on the 2 tasks. |
| **Sunk cost fallacy**  - refers to people’s tendency to continue an activity if they have already invested money, time or effort in it  (Tasks adapted from Arkes and Blumer, 1985) | *Task*  As the CEO of your company, you enroll your employees on finance training course “A”. The total cost of course “A” for the three people is 500.000 HUF. You find out the next week that finance training “B” on the same topic as training “A” would be more useful for your employees. You pay for the total price of training “B”, which is 100.000 HUF. You only realize later on that the two courses are held at exactly the same time. Because both of the courses are experience-based, your colleagues cannot pass on the benefits of the course to other colleagues that did not attend. Which of the courses do you send your colleagues to?  *Answer options*  Send all three colleagues to training "A";  Send two colleagues to training "A", while sending one to "B";  Send one colleague to training "A", while sending two to "B";  * Send all three colleagues to training "B". | *Task*  Your company manufactures audio and lighting equipment. You started the development of a new type of spotlight two years ago. During the two years, the development cost (wages, technology, etc.) has been 70 million HUF, but you need another 30 million HUF for perfecting the product and putting it onto the market, which is expected to happen in a year. If you cannot provide this amount of money, you will have to stop the development, and the project will fail. In the meanwhile, the audio division of your company is also considering development projects. With a 30 million HUF development cost, they would be able to manufacture a new type of loudspeaker within one year. This loudspeaker, according to the forecasts, could be even more profitable on the market than the reflector. The maximum amount of money available for development is 30 million HUF. This means that you can realize only one of the projects. Which project do you decide to finance?  *Answer options*  Spend the whole sum on the development of the spotlight;  * Spend the whole sum on the development of the speaker;  Spend 15 million HUF on each project;  20 million HUF on the development of the speaker, and the rest on the development of the spotlight. |
| **Base rate neglect**  - refers to the tendency of people to neglect statistical base rate information when making decisions  (Tasks adapted from Fong et al., 1986) | *Task/1*  As the Chief Financial Officer of a corporation, you are planning to buy new laptops for the workers of the company. Today, you have to choose between two types of laptops that are almost identical with regard to price and the most important capabilities. According to statistics from trusted sources, type “A” is much more reliable than type “B”. One of your acquaintances, however, tells you that the motherboard of the type “A” laptop he bought burnt out within a month and he lost a significant amount of data. As for type “B”, none of your acquaintances have experienced any problems. You do not have time for gathering more information. Which type of laptop will you buy?  *Answer options*  * Order type “A” laptops only;  Order type “B” laptops only;  50% percent of laptops ordered should be type “A”, 50% percent type “B”;  25% percent of laptops ordered should be type “A”, 75% percent type “B”. | *Task/1*  Because the sales returns were lower than expected, you have to cut back on employees at your company. You have decided to dismiss one of the two salesmen. John achieved a stable average performance in the past few years. The other salesman, Tom, had a poorer performance during the past few years. In the past month, however, Tom displayed surprisingly good performance, whereas John's performance dropped back. You have to make a decision today. Who will you dismiss?  *Answer options*  Dismiss John; *Dismiss Tom.  *Task/2*  You are the HR manager of a company and have two candidates for a sales position: Erik and Adam. Judging by their résumés you come to understand that Erik has better sales performance. In the long term he succeeds in 70% of cases. The practice situation yielded the following result in performance: Eric: 40%; Adam: 70%. Who will you promote?  *Answer options*  *Promote Erik; Promote Adam.  *Correct answer*  Coherently choosing by the base rates on the tasks. |
| **Insensitivity to sample size**  - refers to people’s tendency to disregard the fact that small samples don’t follow the laws of big samples  (Tasks adapted from Tversky and Kahneman, 1974) | *Task*  Your task is to predict the expected income of two of the company's divisions. The company's division “A” is twice as big as its division “B”, but they are identical in all other relevant aspects. Which division is more likely to exceed your income prediction by 30%?  *Answer options*  Division “A”;  * Division “B”;  Chances are the same for both divisions;  Cannot provide an answer from the information given. | *Task*  You work for a large automobile manufacturing company and are responsible for the acquisition of the manufacturing machinery. You have two machines that produce windshield glass. Machine “A” works 5 hours each day, while machine “B” works 10 hours. Both produce the same percentage of substandard glass. Your job is to estimate the substandard/ standard glass ratio. Which of the two machines are more likely to produce an unusually high proportion of substandard glass in the following month?  *Answer options*  * Machine “A”;  Machine “B”;  The chance is the same for both machines;  Cannot be determined from the given information. |
| **Regression to the mean**  - refers to the tendency of people not to take into account that after an extreme value the next value will more probably be closer to the mean  (Tasks adapted from Bazerman, 2005) | *Task*  Your company has two subsidiary companies, “A” and “B”. Although these subsidiary companies are identical with regard to all relevant economic indicators, significantly different incomes were accounted in the past several months. The average income per month is 50,000 dollars for both companies. Please estimate the expected incomes of the next month after reviewing the past incomes.   \|  \| Aug \| Sept \| Oct \| Nov \| \| --- \| --- \| --- \| --- \| --- \| \| A \| 40 M \| 20 M \| 60 M \| 80 M \| \| B \| 20 M \| 80 M \| 60 M \| 40M \|   *Answer options*  “A”: 40,000, “B”: 20,000;  “A”: 90,000, “B”: 30,000;  * “A”: 50,000, “B”: 50,000;  “A”: 60,000, “B”: 60,000. | *Task*  The management of a chain store offers you an advisory position. Your job is to most accurately estimate next year's turnover rate for each store. The development and marketing budget plan for next year will be based on your estimation. The stores are identical with regard to both scale and the products offered; differences in the stores' sales figures are mostly caused by random fluctuation. According to reliable trade forecasts, a 10% increase is expected in this year's total turnover rate. The stores had the following turnover rates in 2011. Please indicate your best forecasts about the stores' turnover rate in 2012.   \|  \| 2012 \| 2013 \| 2013 \| 2013 \| 2013 \| \| --- \| --- \| --- \| --- \| --- \| --- \| \|  \|  \| A \| B \| C \| D \| \| Store 1 \| 22 M \| 24.2 M \| 28.4 M \| 18.4 M \| 24.2 M \| \| Store 2 \| 24 M \| 26.4 M \| 24.2 M \| 30 M \| 24.2 M \| \| Store 3 \| 20 M \| 22 M \| 20 M \| 24.2 M \| 24.2 M \| \| Mean \| 22 M \| 24.2 M \| 24.2 M \| 24.2 M \| 24.2 M \|   *Answer options*  A;  B;  C;  *D. |
| **Covariation detection**  - refers to how people judge whether a component has an effect, with or without taking into account the other elements of the contingency table  (Tasks adapted from Stanovich and West, 1998) | *Task*  Your company sells dietary supplements for athletes. You would like to test the effectiveness of the new NutroX energy slice before putting it on the market, so you test the product on professional runners. What is the least amount of statistical data you need in order to make certain of the effectiveness of the product?  A) The number of runners that consumed NutroX with their performance improving  B) The number of runners that consumed NutroX without their performance improving  C) The number of runners that did not consume NutroX and their performance improved  D) The number of runners that did not consume NutroX and their performance did not improve  *Answer options*  Only information A;  Information A and B;  Information A, B and C;  * Information A, B, C and D. | *Task*  As the new CEO of a financial advisory company. You notice that if you praise your employees when their performance improves, the following month their performance decreases, and if you call them to account after a decrease in performance then sales figures seem to increase.  How would you modify the benchmarking system solely in light your acquired experience in order to improve overall performance?  *Answer options*  Based on my experience I would place accountability as a central part of leadership for constant performance increase;  * I cannot tell whether praise or calling to account have a greater effect on performance based on my experience;  In case of stagnant and decreasing performance I would keep regular and strict accountability as long as the expected performance increase does not occur;  I continue calling employees to account, but only when the performance decreases. |
| **Framing effect**  - refers to the tendency of people to decide differently when the same information is worded differently  (Tasks adapted from Tversky and Kahneman, 1981) | *Task/1*  You would like to apply for funding for the development of a new technology. A consulting firm offers to write the application for you. The firm is one of the more expensive consulting firms. According to the information available to you, this firm loses applications in 5 out of 20 cases. Based on this information, would you accept the company's offer to write an application?  *Task/2*  You are the owner of a rural hotel. To repair and expand the building, you would like to apply for funding. A consulting firm offers to write the application for you. The firm is one of the more expensive consulting firms. According to the information available to you, the firm wins applications in 15 out of 20 cases. Based on this information, would you accept the company's offer to write the application?  *Answer options for both tasks*  Please indicate on a 4-point scale, how willing you would be to hire the consulting firm.  (1 - Definitely not hire; 4 - Definitely hire)  *Correct answer*  Coherent choices on the tasks. | *Task/1*  You would like to sue one of your former clients because he owes 6 million HUF to your company. The company offers to settle the case out of court. If you accept this arrangement, you will be paid 2 million HUF. However, if you decide to go to court, according to the forecast of your lawyers, you have a 33% chance of winning the lawsuit and getting back the total amount of money within one year.  *Task/2*  One of your subcontractors announces that he got into a difficult financial situation. He now owes 6 million HUF to your company. According to his offer, he would pay you immediately, but he definitely will not be able to pay back 4 million HUF out of the 6 million HUF. If you decline this offer and instead decide to wait, you will have a 33% chance that you do not lose any of your assets, but you will have a 67% chance that the subcontractor cannot pay at all should his company go bankrupt. What is your decision?  *Answer options for both tasks*  Please indicate on a 4-point scale, how willing you would be to accept the offer.  (1 - Definitely not hire; 4 - Definitely hire)  *Correct answer*  Coherent choices on the tasks. |
| **Anchoring bias**  - refers to that people tend to adjust their decision to the first information they hear  (Tasks adapted from Tversky and Kahneman, 1974) | *Task/1*  Estimating the population of Belgium among low anchors.  *Task/2*  Estimating the population of Belgium among high anchors.  *Answer options*  Based on their first choice, different values were presented.  *Correct answer*  Choosing the same value consistently on the tasks. | *Task/1*  Estimating the average price of 1 kg of sugar among low anchors.  *Task/2*  Estimating the average price of 1 kg of sugar among high anchors.  *Answer options*  Based on their first choice, different values were presented.  *Correct answer*  Choosing the same price consistently on the tasks. |
| **Overconfidence bias**  - refers to the tendency of people to perceive their ability as better than it actually is | *Tasks*  You will now have to answer a series of questions regarding economic indicators of Hungary.  Please choose the answer you believe correct for the respective question amongst the options!  How many large companies (total number of staff is higher than 250) are there currently in Hungary (2012)?  What is the current gross minimal wage for workers with a high school diploma?  What is current minimum pension?  What was the rate of unemployment in 2011 (percentage)?  What is the per capita GDP in Hungary (2011)?  How much does 1 kg of sugar cost on average?  What is the average gross national income?  How many registered employees are there in Hungary (2012)?  How many registered companies are there in Hungary (2012)?  How many registered companies are there in Budapest (2012)?  How many questions do you think you've answered correctly?    *Scoring*  Perceived accuracy – Real accuracy | *Task*  You will now have to answer a series of questions regarding economic indicators of Hungary.  Please choose the answer you believe correct for the respective question amongst the options!  What is the total length of highway built in Hungary (km)?  What was the total national debt of Hungary (billion HUF)?  What was the domestic inflation in September 2012?  What is population of Budapest?  How many “A category” stocks are there on the Hungarian Stock Exchange?  How big is the social contribution tax?  What is the gross minimum wage for workers with a graduate diploma?  How many state recognized higher education institutes are there in Hungary currently?  How many questions do you think you've answered correctly?    *Scoring*  Perceived accuracy – Real accuracy |
| **Additional question** |  | Could you recall any instance when you made a decision differently, because of the training?  *Answer options*  Yes;  No. |

*Note.* Correct answers are indicated by asterisk if not stated otherwise.

## Supplementary Tables - training materials

**Supplementary Table 2. Training materials for the awareness and analogical training.**

|  | **Real life example** | **Avoidance techniques** |
| --- | --- | --- |
| **Outcome bias** | A story about how physician practice should be evaluated based on the risks and gains of the given procedure, not on the outcome. | (1) “Base the rewards (of employees) on the quality of the decisions, not on the outcomes!”  (2) “Have people who are the least motivated for a good outcome evaluate your projects!”  (all based on Mowen, 1992) |
| **Sunk cost fallacy** | A story about how one should leave the theatre if she is not enjoying the play, but only staying because it has already been paid for. | (1) “Convert monetary expenses into temporal expenses!”  (2) “Look at the ‘Big Picture’!”  (3) “The past doesn’t matter.”  (all based on (Belsky and Gilovich, 2010) |
| **Base rate neglect** | A story about how people, upon purchasing a product, should base their decision on the means of other customers’ ratings, rather then on the salient stories of their friends. | (1) “Filter salient information!”  (2) “Convert to frequencies!”  (all based on Sedlmeier and Gigerenzer, 2001) |
| **Insensitivity to sample size** | A story on how Coca-Cola made a big campaign mistake with a new taste, because they based their decision on the opinion of very few people. | (1) “Anything can happen on a small sample…”  (Tversky and Kahneman, 1971) |
| **Regression to the mean** | A story about how trainers think it is good practice to base their coaching on punishing trainees when they perform poorly and reward them when they perform best. | (1) “Observed behavior = Real ability + Chance”  (Harrison and Bazerman, 1995) |
| **Covariation detection** | A story about testing the effectiveness of an imaginary energy bar based on an experimental and a control group. | (1) “Try to explore the contingency table!”  (Hattori and Oaksford, 2007) |
| **Framing effect** | A story about an opinion-poll, where responders’ answers differed based on whether they were asked if they support the forbidding of anti-democratic speeches, or not allowing them. | (1) “Try to recognize alternative frames!”  (Russo and Schoemaker, 1989)  (2) “Try to reframe the situation!”  (Hammond et al., 1998) |
| **Anchoring bias** | A story of the experiment where people were asked to rate how long a blue whale is, after presenting them with either high or low anchors. | (1) “Try to find an opposite anchor!”  (Whyte and Sebenius, 1997)  (2) “Bootstrapping”  (Larrick, 2004) |
| **Overconfidence bias** | A story about how people usually think that their start-up will not fail despite the fact that most of them will. | (1) “How would I know if I’m wrong?”  (Hammond et al., 1998)  (2) “The predictive power of past behavior.”  (Buehler et al., 1994) |
| **Planning fallacy** | The story of how the plans of the construction of the Sydney Opera House and the actual implementation differed in financial and temporal terms. | (1) “Try to find a reference group!”  (Buehler et al., 2002)  (2) “Try to separate the task into sub-phases!”  (Kruger and Evans, 2004) |

**Supplementary Table 3. Training materials for the analogical training.** Participants received a paper-based “Worksheet” at the beginning of the training, with the different tasks printed on them.

|  | **Task** |
| --- | --- |
| **Outcome bias** | *Task 1* (based on Baron and Hershey, 1988)  The participants had to evaluate how good the decision was in the following story where half of the participants received it with a positive outcome and the other half received it with a negative outcome.  *Description*  *Cheese-space* is a local start-up which plans to build a web-site where people can share their own recipes. The specialty of the site is that one can search by ingredients. For the site to be successful and to gain income from advertisements, first they need to gather recipes from amateurs and professional chefs. To cover the expenses of starting the firm they need to find an investor. After several rejections, investors of Lotus group decided to invest several million HUF in the project.  *Outcome A*  After two years of work and more expenses than planned the project became successful and the investors earned a large profit.  *Outcome B*  After two years of work and more expenses than planned the project was unsuccessful and the investors lost a large amount of money.  *Task 2*  The same structure as Task 1, but with a different story.  *Group discussion for Task 1 and Task 2*  Demonstrating the outcome bias by comparing group differences in decision evaluation.  *Task 3* (adapted from Myers, 2004)  Tap the rhythm of a well-known tune to your partner, and estimate how long it would take for them to figure out what it is.  *Group discussion for Task 3*  It is hard to estimate how long it would take knowing the outcome. |
| **Sunk cost fallacy** | *Task 1* (based on Bazerman, 2005)  You think it is a good idea to buy MOL stocks and you buy 2 million HUF worth. You also think OTP stocks are good, but you only buy 0.5 million HUF worth.  Right after the purchase the rate of OTP stocks starts to raise, MOL stocks starts to decline.  Suddenly you are in a need of 1 million HUF and you are thinking of selling one of your stocks. Which one would you sell?  - 1 million HUF worth of OTP stock that you bought for 0.5 million; or  - 1 million HUF worth of MOL stock that you bought for 2 million.    *Task 2* (based on Staw, 1976)  In the Vietnam War more than 100 000 American and Australian people died, while only 4400 Vietnamese. The number of American and Australian soldiers sent into combat increased as the years passed, even though they continued to lose more soldiers and suffered more loss than their opponents.  Why would you think this might have happened?  *Task 3*  You are the manager of a pharmaceutical company. Your boss wants you to aid her decision on whether she should support the development of a new drug. The question is whether to stop the development now or move forward with the production.  The following list consists of the factors you have information on. Please indicate which factors you think are irrelevant for the decision.  (List not presented here; the only irrelevant factor is “the expenses of development so far”.)  *Group discussion for Task 1, Task 2 and Task 3*  Demonstrating the sunk cost fallacy through the discussion of each task. |
| **Base rate neglect** | *Task 1* (based on Lichtenstein et al., 1978)  Which one do you think has the highest incidence per year?   - Homicide; - Suicide.   *Task 2*  Which one do you think has the highest incidence per year?   - The combined number of homicide, suicide and death from car crashes; - Cancer.   *Task 3* (based on Tversky and Kahneman, 1974)  Istvan is mostly shy and introverted, but always helpful. He is not very much interested in people and matters of the world. He is modest and orderly. Details and order are very important to him.  What do you think is Istvan’s occupation?   - Courier; - Physician; - Librarian.   *Group discussion for all Tasks*  Demonstrating base rate neglect through the discussion of the correct answers. |
| **Insensitivity to sample size** | *Task* (Tversky and Kahneman, 1974)  In a town there are two hospitals. The maternity ward of Hospital ‘A’ is twice as large as Hospital ‘B’.  In which hospital is it more probable that 40% of babies born on a given day are girls?  *Group discussion*  Demonstrating insensitivity to sample size through the discussion of the correct answers. |
| **Regression to the mean** | *Task*  Find structural analogies between two pairs of the stories below.  *Story 1* (based on Kahneman, 2011)  Antal is an air traffic controller. He says that when he used to compliment the pilots after a smooth performance of a difficult maneuver, they usually performed worse the next day. He also often scolded them for their mistakes, then they usually performed better the next time. Antal, based on his experience thinks that for the performance of the pilots to improve he has to punish them for their mistakes rather than giving compliments.  *Story 2*  Alfa Power Ltd. produces power bars and the management wanted to know the effectiveness of their new product. The employee who conducted the research argues that they should distribute the new product because they found 80 athletes who performed better after the consumption of the new energy bar for two weeks. The management wasn’t very convinced by the results.  *Story 3*  Students of Oxford University are convinced that they are better in mathematics than the students of Cambridge University. They underpin the statement with the fact that in the national measures 30 Cambridge student failed, while this number for Oxford students was 15. Students of Cambridge think that this rather proves that Oxford students cannot count.  *Story 4*  An international soccer club team performed unprecedentedly bad lately, so the management fired the coach. Many people questioned if this was the right decision, but right in the first match after the new coach started the team performed better than last time. The management and the owner thought that they made the right decision, so they proposed a long-term contract to the new coach.  *Group discussion*  Demonstrating Regression to the mean and Covariation detection through the structural pairs (Stories 1, 4 and 2, 3). |
| **Covariation detection** |  |
| **Framing effect** | *Task* (based on Sebenius, 2001)  Bargaining task, with the instruction of: “Try to reach the best agreement with your partner!”  *Role 1.*  In 1912, Theodor Roosevelt during the heat of the presidential election visited each and every state. His campaign managers planned that before each appearance of their candidate, leaflets were to be distributed with Roosevelt’s photo on them. When 3 million copies were already printed for the campaign, one activist noticed the line ‘Mofflett Studio, Chicago’ written under the photo. Since the photo was copyrighted, they would have to pay 1 dollar per copy to the photography studio. If they would decide not to use the leaflets, Roosevelt might lose the election.  Your role is to act as the campaign manager. Your task is to meet the director of the Mofflett studio and reach an agreement. The studio does not yet know that you want to use their photo. Now turn to your partner and try reaching a favorable agreement.  *Role 2.*  It is 1912, and you are the CEO of Mofflett Photography Studio, Chicago. Soon you will receive a business offer. Try to reach an agreement most profitable for your studio.  *Group discussion*  The original story, where the campaign manager made the studio pay for the “privilege” of using their picture. |
| **Anchoring bias** | *Task 1/a* (based on Tversky and Kahneman, 1974)  1×2×3×4×5×6×7×8 = ?  *Task 1/b*  8×7×6×5×4×3×2×1 = ?  *Task 2/a* (based on Frederick and Mochon, 2012)  What do you think is the population of Turkey? Greater, or smaller than 6 million?  *Task 2/b*  What do you think is the population of Turkey? Greater, or smaller than 600 million?  *Group discussion for Task 1 and Task 2*  Demonstrating group mean differences for the different tasks. |
| **Overconfidence bias** | *Task*  6 general knowledge question quiz, with gambling opportunity.  *Group discussion*  Demonstrating the overconfidence effect with most people losing all their token money. |
| **Planning fallacy** | *Task* (based on Forsyth and Burt, 2008)  “Estimate how long cooking goulash soup would take in minutes!”; on half of the groups’ worksheet five sub-phases were also printed.  *Group discussion*  Demonstrating planning fallacy by comparing estimation differences between the groups. |

## Supplementary Tables – Results

**Supplementary Table 5. Percentage of correct answers across experimental conditions for both pre- and post-training tests.**

|  | Control | | Awareness | | Analogical | |
| --- | --- | --- | --- | --- | --- | --- |
|  | Before test | After test | Before test | After test | Before test | After test |
| Anchoring bias | 57.4% | 75.9% | 46% | 78% | 50% | 76% |
| Framing effect | 44.4% | 59.3% | 44% | 58% | 46% | 48% |
| Overconfidence bias | 33.4% | 29.6% | 40% | 20% | 54% | 34% |
| Sunk cost fallacy | 24.1% | 51.9% | 28% | 48% | 38% | 48% |
| Outcome bias | 31.5% | 51.9% | 24% | 64% | 36% | 60% |
| Base rate neglect | 35.2% | 38.9% | 36% | 52% | 42% | 64% |
| Insensitivity | 16.7% | 9.3% | 18% | 6% | 18% | 30% |
| Covariation detection | 14.8% | 13% | 16% | 46% | 34% | 46% |
| Regression to the mean | 22.2% | 11.1% | 34% | 20% | 20% | 18% |

**Supplementary Table 6. Means and standard deviations of the Statistical biases composite scores.**

|  | Control | | Awareness | | Analogical | |
| --- | --- | --- | --- | --- | --- | --- |
|  | Before test | After test | Before test | After test | Before test | After test |
| Mean | 0.89 | 0.72 | 1.04 | 1.2 | 1.14 | 1.58 |
| SD | 0.9 | 0.74 | 0.97 | 0.97 | 0.9 | 1.03 |

**Supplementary Table 7. Cohen’s d effect sizes between pre- and post training test scores for the Statistical biases.**

|  | Statistical biases composite | Insensitivity to sample size | Base rate neglect | Regression to the mean | Covariation detection |
| --- | --- | --- | --- | --- | --- |
| Control | -0.20 | -0.22 | 0.08 | 0.3 | 0.05 |
| Awareness | 0.17 | -0.37 | 0.32 | 0.32 | 0.59 |
| Analogical | 0.45 | 0.28 | 0.44 | 0.05 | 0.24 |

1. **References**

Arkes, H. R., and Blumer, C. (1985). The psychology of sunk cost. *Organ. Behav. Hum. Decis. Process.* 35, 124–140. doi:10.1016/0749-5978(85)90049-4.

Baron, J., and Hershey, J. C. (1988). Outcome bias in decision evaluation. *J. Pers. Soc. Psychol.* 54, 569–579. doi:10.1037/0022-3514.54.4.569.

Bazerman, M. (2005). *Judgment in managerial decision making*. Hoboken, NJ: John Wiley & Sons.

Belsky, G., and Gilovich, T. (2010). *Why smart people make big money mistakes and how to correct them: Lessons from the life-changing science of behavioral economics*. New York, NY: Simon and Schuster.

Buehler, R., Griffin, D., and Ross, M. (1994). Exploring the “planning fallacy”: Why people underestimate their task completion times. *J. Pers. Soc. Psychol.* 67, 366–366. doi:10.1037/0022-3514.67.3.366.

Buehler, R., Griffin, D., and Ross, M. (2002). “Inside the planning fallacy: The causes and consequences of optimistic time predictions,” in *Heuristics and biases: The psychology of intuitive judgment*, eds. T. Gilovich, D. Griffin, and D. Kahneman (New York, NY: Cambridge University Press), 250–270.

Fong, G. T., Krantz, D. H., and Nisbett, R. E. (1986). The effects of statistical training on thinking about everyday problems. *Cognit. Psychol.* 18, 253–292. doi:10.1016/0010-0285(86)90001-0.

Forsyth, D. K., and Burt, C. D. B. (2008). Allocating time to future tasks: The effect of task segmentation on planning fallacy bias. *Mem. Cognit.* 36, 791–798. doi:10.3758/MC.36.4.791.

Frederick, S. W., and Mochon, D. (2012). A scale distortion theory of anchoring. *J. Exp. Psychol. Gen.* 141, 124–133. doi:10.1037/a0024006.

Hammond, J. S., Keeney, R. L., and Raiffa, H. (1998). The hidden traps in decision making. *Harv. Bus. Rev.* 76, 47–58.

Harrison, J. R., and Bazerman, M. H. (1995). “Regression to the mean, expectation inflation, and the winner’s curse in organizational contexts,” in *Negotiation as a social process: New trends in theory and research*, eds. R. M. Kramer and D. M. Messick (Thousand Oaks, CA: Sage Publications, Inc), 69–94.

Hattori, M., and Oaksford, M. (2007). Adaptive Non-Interventional Heuristics for Covariation Detection in Causal Induction: Model Comparison and Rational Analysis. *Cogn. Sci.* 31, 765–814. doi:10.1080/03640210701530755.

Kahneman, D. (2011). *Thinking, fast and slow*. New York, NY: Farrar, Straus and Giroux.

Kruger, J., and Evans, M. (2004). If you don’t want to be late, enumerate: Unpacking reduces the planning fallacy. *J. Exp. Soc. Psychol.* 40, 586–598. doi:10.1016/j.jesp.2003.11.001.

Larrick, R. P. (2004). “Debiasing,” in *Blackwell handbook of judgement and decision making*, eds. D. J. Koehler and N. Harvey (Malden, MA: Blackwell Publishing Ltd), 316–337.

Lichtenstein, S., Slovic, P., Fischhoff, B., Layman, M., and Combs, B. (1978). Judged frequency of lethal events. *J. Exp. Psychol. [Hum. Learn.]* 4, 551–578. doi:10.1037/0278-7393.4.6.551.

Mowen, J. C. (1992). The time and outcome valuation model: Implications for understanding reactance and risky choices in consumer decision making. in *NA - Advances in Consumer Research Volume 19*, eds. J. F. Sherry and B. Strenthal (Provo, UT: Association for Consumer Research), 182–189.

Myers, D. G. (2004). *Intuition: Its powers and perils*. New Haven, CT: Yale University Press.

Russo, J. E., and Schoemaker, P. J. H. (1989). *Decision traps: Ten barriers to brilliant decision-making and how to overcome them*. New York, NY: Doubleday New York.

Sebenius, J. K. (2001). Six habits of merely effective negotiators. *Harv. Bus. Rev.* 79, 87–97.

Sedlmeier, P., and Gigerenzer, G. (2001). Teaching Bayesian reasoning in less than two hours. *J. Exp. Psychol. Gen.* 130, 380–400. doi:10.1037/0096-3445.130.3.380.

Stanovich, K. E., and West, R. F. (1998). Individual differences in rational thought. *J. Exp. Psychol. Gen.* 127, 161–188. doi:10.1037/0096-3445.127.2.161.

Staw, B. M. (1976). Knee-deep in the big muddy: a study of escalating commitment to a chosen course of action. *Organ. Behav. Hum. Perform.* 16, 27–44. doi:10.1016/0030-5073(76)90005-2.

Tversky, A., and Kahneman, D. (1971). Belief in the law of small numbers. *Psychol. Bull.* 76, 105–110. doi:10.1037/h0031322.

Tversky, A., and Kahneman, D. (1974). Judgment under uncertainty: Heuristics and biases. *Science* 185, 1124–1131. doi:10.1126/science.185.4157.1124.

Tversky, A., and Kahneman, D. (1981). The framing of decisions and the psychology of choice. *Science* 211, 453–458. doi:10.1126/science.7455683.

Whyte, G., and Sebenius, J. K. (1997). The Effect of Multiple Anchors on Anchoring in Individual and Group Judgment. *Organ. Behav. Hum. Decis. Process.* 69, 74–85. doi:10.1006/obhd.1996.2674.
